# Neurophysiology, Neuro-Immune Interactions, and Mechanobiology in Osteopathy in the Cranial Field: An Evidence-Informed Perspective for a Scientific Rationale

**DOI:** 10.3390/healthcare11233058

**Published:** 2023-11-28

**Authors:** Nicola Barsotti, Alessandro Casini, Marco Chiera, Christian Lunghi, Mauro Fornari

**Affiliations:** 1 Centre for Osteopathic MEdicine (COME) Foundation, 65121 Pescara, Italy; nicola.barsotti@accademiamibes.it (N.B.); alessandroxcasini@gmail.com (A.C.); marco.chiera.90@gmail.com (M.C.); 2 Società Italiana di Psiconeuroendocrinoimmunologia (SIPNEI), 00100 Roma, Italy; 3 Collegio Italiano di Osteopatia (CIO), 43123 Parma, Italy; c.i.o.maurofornari@gmail.com; 4 BMS Formation, 75116 Paris, France; 5 Osteopatia Lunghi-Baroni Private Practice, 00146 Rome, Italy

**Keywords:** manipulation, osteopathic, osteopathy in the cranial field, manual therapy, touch, mechanotransduction, trigeminal nerve, cranial sutures

## Abstract

(1) Background: Osteopathy in the cranial field (OCF) is a distinctive approach within osteopathy, focusing on the cranial region’s manual manipulation. Nevertheless, OCF fosters doubt in the scientific community because it refers to out-of-date models. This perspective paper critically analyzes the current knowledge in the fields of neurophysiology and mechanobiology to propose an evidence-informed rationale for OCF. (2) Methods: The reporting framework used in the current perspective article obeyed the guidelines for writing a commentary. (3) Results: The article’s main findings focus on the tactile stimulation of exocranial receptors and their implications in the management of craniofacial algic-dysfunctional syndromes implementing OCF. (4) Conclusions: By establishing an evidence-based rationale for OCF, this research aims to guide future directions in OCF and contribute to a more patient-centered and effective approach to health and wellbeing.

## 1. Introduction

Osteopathy is a person-centered approach, particularly focused on the bidirectional relationship between the body’s structure/function and its adaptation to environmental demands [1]. Through osteopathic evaluation, osteopathic manipulative treatment (OMT), and patient engagement [2], osteopathy aims to restore health by integrating evidence-informed strategies for managing specific clinical contexts, with personalized approaches tailored to individual needs and preferences [1].

The manipulative approach, according to osteopathic paradigms, centers on identifying functional disturbances associated with regions of the body, known as somatic dysfunctions (SDs) [3,4,5]. Osteopaths consider SDs as an interface for customizing the biological and psychological effects of touch [1]. Touch serves as the primary non-verbal clinical communication tool used by osteopaths to detect SDs collaboratively with the patient, and to perform specific manipulative techniques, selected through shared decision making [1].

While various systematic reviews have highlighted the need for more rigorous clinical studies to generalize the results obtained with OMT [5,6,7], several studies have shown positive effects in supporting individuals with musculoskeletal [8,9], neurological [6], and inflammatory disorders [7], across different life stages, from neonatal [10,11] to advanced age [12].

One approach traditionally considered distinctive in osteopathic practice is osteopathy in the cranial field (OCF) [13,14]. The cranial approach is described by the World Health Organization as one of the core osteopathic competencies [15]. Consequently, osteopathic training includes anatomical study of the cranial bones, their sutures, and their intrinsic mobility. According to W.G. Sutherland’s view, this intrinsic mobility is termed the primary respiratory mechanism, comprising five components: central nervous system motility, cerebrospinal fluid fluctuation, reciprocal tension membranes, cranial bone mobility, and involuntary motion of the sacrum between the ilium [13,16,17,18,19,20,21].

In practice, OCF involves manual contact by the therapist on the patient’s cranial skin to gather information for osteopathic clinical reasoning and to detect cranial SDs [13], which represent neurologically active areas associated with specific biological functions and psychological perceptions [1].

In recent years, several studies, including experiments on animal models [21,22] and on healthy subjects and those with functional disorders [23], have evaluated the impact of OCF on various aspects of health (Table 1). Studies conducted on healthy subjects have employed quantitative electroencephalographic evaluation of alpha waves [16] and assessment of endocrine and autonomic regulation under mental stress [24], demonstrating positive effects on autonomic nervous system (ANS) activity [19]. A case report on a single adult patient showed improvement in symptoms associated with whiplash following OCF treatments [25]. A clinical trial observed that integrated OCF–OMT treatments positively affected gait kinematics in patients with Parkinson’s disease [26], whereas a systematic review concluded that OCF treatments can improve pain and functionality in patients with chronic pain [27]. A scoping review published in 2022 also reported that a combination of osteopathic techniques targeting various body regions, including the cranial area, can help reduce the intensity and frequency of migraine headaches and acute medication use, thereby improving the quality of life for patients with migraines [28].

Despite these experimental findings, the research quality in this field appears weak, and the evidence supporting OCF treatment remains scientifically limited and inconsistent [18]. Consequently, a part of the osteopathic community has recently asserted that cranial approaches should not be employed in contemporary osteopathic practice due to outdated models known to be misleading, generate empirical observations, and instill mistrust in the profession [37]. The reliance on dated theories often constitutes the sole rationale for cranial approaches, as the methodology and treatment principles for the cranial region date back to the early 20th century. Sutherland’s model, in particular, is inspired by theories that are no longer supported by the current knowledge of physiology [38], such as those explained in Emanuel Swedenborg’s anatomical studies in the 1700s [17]. Nonetheless, recent scientific evidence suggests that it is the contractile fibers in the cranial muscles that may primarily contribute to cranial loading and deformation [38]. Beyond the aforementioned criticisms, such evidence would make the head region, like the rest of the body, amenable to touch-based approaches like osteopathy [38].

To date, there is no shared theoretical framework supporting an evidence-informed osteopathic approach. Previously used models do not support OCF, and available evidence does not support either the diagnostic reliability of procedures used in OCF or its therapeutic efficacy [18].

However, emerging clinical findings from research conducted thus far show encouraging results and align with patient satisfaction after receiving osteopathic treatments focusing on the whole body [39], including the head area [40]. Therefore, in accordance with the principles of evidence-based medicine (EBM), it is not appropriate to dismiss an approach in the presence of positive patient-reported experiences [38].

Osteopaths are thus facing an ethical dilemma concerning EBM [37]. On one hand, the currently available evidence suggests refraining from OCF treatments [18]. On the other hand, the personal or collective experience of successfully treating limited subgroups of patients with specific conditions suggests the acceptance of applying such treatment despite limited evidence to support its use [40].

Therefore, it would be sensible to involve informed patients in the final decision-making process rather than relying solely on healthcare practitioners [37]. This approach would likely be more aligned with efforts to place patients at the center of EBM, incorporating patient values into clinical decision making [41].

In promoting a person-centered osteopathy, which is culturally sensitive to diverse socio-cultural health assumptions and informed by evidence, it is essential to propose ideas that can stimulate internal debate within the professional community and encourage research agendas that address open questions regarding the practice models linked to traditional principles.

By examining the available literature on thermo–mechano–chemical stimulations of the craniofacial region, specifically on cranial sutures, and their impact on craniofacial algic-dysfunctional syndromes [29,42,43,44], the authors will outline a rationale for evidence-informed cranial osteopathy.

Therefore, the aim of this article is to critically analyze the current knowledge on the tactile stimulation of exocranial receptors, and their related endocranial and secondary systemic effects, to consider their implications in the treatment of different algic-dysfunctional disorders, and to establish a potential scientific rationale for OCF. An evidence-based theoretical framework could indeed promote the development of novel approaches for research and clinical practice in the field of OCF, as well as the broader domain of manual therapies in osteopathy.

The methods section provides a brief description of the strategies implemented to conduct the literature search necessary to carry out the critical analysis outlined above; the results section in the present paper covers the in-depth analysis of the literature relevant for defining a sound scientific rationale for OCF. The discussion section then resumes the main findings from the literature review and presents their implications for the clinical practice of osteopathic medicine; in this section, the limitations regarding the present paper and the actual state of osteopathic research, as well as the future directions needed to strengthen both osteopathic research and practice, are also discussed.

## 2. Methods

The current review’s reporting framework adhered to the guidelines for designing a commentary [45]. To address the aim of the present paper, the authors considered the following 2 questions relevant for the osteopathic practice of OCF:

1. Is there a documented relationship between nociception in the pericranial myofascial tissue and nervous system excitability?
2. Is the innervation of the cranial sutures, the mechanoreceptors responsive to touch, and the sensory fibers involved in the transmission of nociceptive signals (i.e., headache), from the intracranial and extracranial blood vessels?

The theoretical framework for the current review was developed by a working group of experts with at least 10,000 h of professional experience in education and scientific research and/or in clinical osteopathic practice. More specifically, the framework was the result of a brainstorming process based on clinical observation and the best available evidence.

To identify eligible articles that would inform the current review, a literature search was performed between October and April 2022 in the following databases: MEDLINE (PubMed), EMBASE, and Google Scholar. The search terms (i.e., keywords: cranial sutures; mechanoreceptors; mechanotransduction; touch; trigeminal nerve; headache; manipulation, osteopathic; osteopathy in the cranial field; manual therapy) were adapted for each database, and suitable subheadings were used for each database searched. The search was limited to papers published in English. No limits were applied to the study design, population, study outcome, or date of publication. Reference lists from the articles were also searched, and a snowball procedure was used to identify more relevant articles. To avoid placing any restrictions on the review and to capture the entire range of information about the topic, validity and quality assessments were not performed.

## 3. Results

To provide critical analysis of the scientific literature about cranial sutures and their relevance for osteopathic clinical practice, the selected results from the literature search were reported and grouped by pertinence into the five subsections described below.

### 3.1. Mechanoreception Sensitive to Touch at the Craniofacial Level

The sensory and nociceptive innervation of pericranial tissues (skin, fascia, tendons, and muscles), periosteum, cranial vault bones, and meninges is provided by a dense network of nerve fibers originating from the trigeminal nerve [46,47,48,49,50,51] and nerves exiting the dorsal root ganglia (DRG) at the C2–C3 level [44,47]. The receptors present in the pericranial region include innervated cells (Merkel discs), connective tissue corpuscles encapsulating axon terminals (Pacini, Ruffini, and Meissner corpuscles), and free nerve endings (FNEs) [52]. Additionally, epidermal keratinocytes play a significant role, especially in nociceptive stimulation [53], along with connective tissue fibroblasts [54].

The literature indicates that these receptor sites are mechanoreceptors capable of converting mechanical stimuli into biochemical and electrical signals. This signal transformation is ensured by the presence of a series of specialized proteins, forming mechanically activated ion channels (MAICs) on the cell membrane [55]. Among these proteins, a prominent role is played by transient receptor potentials (TRPs) [56], acid-sensing ionic channels (ASICs) (essential for nociception), and, notably, Piezo channels [57], all of which mediate various neural signaling processes involved in temperature regulation, pressure sensing, pH modulation, nociception, and, consequently, central pain generation [58].

At the cutaneous level, mechanical stimuli activate these ion channels, including TRP vanilloid (TRPV) 1, TRP melastatin (TRPM) 8, and Piezo 1 and 2 [49]. This, in turn, leads to the local release of neuropeptides, such as substance P (SP) and calcitonin gene-related peptide (CGRP), which can initiate inflammatory processes both locally and at the level of the dura mater [49,50,59].

In addition to being activated by mechanical stimuli, TRPs also function as thermal receptors. Different classes of these receptors respond to various temperature thresholds. For instance, while TRPV3 receptors are activated within the temperature range of 32 °C to 39 °C, TRPV4 receptors, which are sensitive to mechanical strains, are activated at temperatures between 27 °C and 34 °C, and TRPM8 receptors at temperatures in the range of 25–34 °C [60,61], which corresponds to typical skin temperatures.

Each of the aforementioned mechanoreceptors are connected to specific nerve fibers capable of transmitting information from the periphery to the central nervous system (CNS) [53,62,63,64]. These fibers can be of type Aβ, Aδ, and C [65], with Aβ myelinated fibers and C fibers, particularly C-tactile (CT) fibers, being the most represented at the cutaneous level [66,67].

The data on young adults demonstrate that the innervation of sensory fibers at the cranial level is denser in the posterior head and neck region (approximately 17 units/cm^2^), while it appears to be relatively equal to the rest of the body in other areas [66]. The face is densely innervated, particularly in the region around the mouth and lips, with an estimated 43,000–46,000 tactile afferents innervating the hairy skin of the face and lips [66]. However, the innervation density is not uniform across the entire face: approximately 48 units/cm^2^ in the forehead, eyes, and nose (V1), 67 units/cm^2^ in the central part of the face (V2), and 84 units/cm^2^ for the lower lip, chin, jaw, and area around the ears (V3) [66]. The cell bodies of these fibers at the cranial level are found in the DRG of C2–C3 and the sensory ganglia of the trigeminal nerve [68].

A significant number of afferent sensory fibers (types C and Aδ) are also present in the cranial periosteum, making them sensitive to mechanical stimulation and nociception [48]. These fibers pass through the cranial sutures, which are fibrous tissue joints known as syndesmoses [69], before spreading into the dura mater [50,70]. TRPV2 ion channels, mechano-nociceptors in the somatosensory system, seem to play a crucial role for these fibers [71].

### 3.2. Exo-Endocranial Connections Mediated by Mechanoreceptor Systems Sensitive to Touch, Distributed at the Pericranial Level and Concentrated in the Sutures

Nerve fibers originating from connective fasciae, tendon junctions, pericranial muscles (with the highest density of nerves found in the temporal and upper nuchal muscles, where their innervation overlaps with the occipital nerves), and the temporomandibular joint capsule, exocranial nerve fibers extend to the periosteum, running close to the arterial blood vessels [30]. From the periosteum, this neural network continues its path inside the cranium, connecting with the endocranial dura mater and influencing its function [46,48,49,50].

Studies conducted on adult mice, confirmed in many areas in humans as well [46], have highlighted the pathways through which these nerves pass from the external to the internal cranial vault, and vice versa (Appendix A): (1) cranial vault sutures, considered the main passage route [43,48,50,59,70]; (2) cranial emissary canals [43,48,50,59,70]; (3) cervico-occipital canals [44].

While the pathways are not precisely defined, nerve fibers have been found in the bone marrow of cranial vault bones, running through various planes of space. These fibers were observed in proximity to leukocytes, osteoblasts, red blood cells, megakaryocytes, and capillary endothelial cells [43,48,50,59,70].

Once these exocranial nerve fibers enter the cranial cavity, they branch out and spread across the whole dura mater. Despite considerable interindividual variations, the dural nerves follow three main routes to converge in the trigeminal nerve [72] and one route that leads to the spinal cord through the C2–C3 spinal nerves [44,47] (Appendix B): (1) a pathway to the anterior cranial fossa, converging in V1 [43,48]; (2) a pathway to the middle cranial fossa, converging in V2–V3 [50]; (3) a postero-superior pathway, carrying the fibers of the transverse sinus, torcular, tentorium, and posterior falx to V1, following the tentorial nerve [73]; (4) a postero-inferior pathway, from the sub-tentorial dura mater, directed towards the DRG at the C2–C3 level to connect with the C2–C4 spinal cord segments [44].

The three branches of the trigeminal nerve reunite at the Gasserian ganglion and, subsequently, reach the trigeminal nuclei in the brainstem. In this regard, it has been demonstrated that: (1) the areas involved in the transmission and processing of nociception at the craniofacial and dural levels are localized in the caudal spinal trigeminal nucleus [46,74]; (2) the areas involved in the transmission and processing of the epicritic tactile sensitivity of the face and scalp are localized in the principal sensory trigeminal nucleus within the pons [72].

### 3.3. Systemic Responses Induced by Exo-Endocranial Communication through the Mediation of the Trigeminal System and the Central Autonomic Network

The activation of the trigeminal nuclei through exocranial stimulation initiates complex pathways that contribute to the maintenance of homeostasis, with the particular involvement of the central autonomic network (CAN). Various types of studies have revealed connections between the trigeminal sensory nuclei and other nuclei in the brainstem, as well as with limbic and cortical structures [72,75,76,77]. The primary trigeminal pathway transmits sensory information from the face and head to the thalamic nuclei and, then, to the sensory cortex [72]. However, there are also other pathways through which the trigeminal sensory nuclei integrate somatic information from the face into the extra-trigeminal structures involved in autonomic and higher functions (cognitive, emotional, behavioral) [72,78,79].

The trigeminal system is interconnected with the CAN, which represents the network of self-regulation and homeostasis par excellence [78,80]. Within this network, the trigeminal system is also linked to the rostral ventrolateral medulla (RVLM) in the brainstem and the nuclei of the vagus nerve, which contribute to the regulation of the sympathetic and parasympathetic activity at the systemic level, respectively [79]. These findings demonstrate that, although the trigeminal nerve does not contain autonomic fibers itself, it participates in reflexes involving the CAN for the maintenance of internal homeostasis.

In this regard, one of the research fields that is contributing significantly to clarifying the functions of the trigeminal system is the study of trigeminal nerve stimulation (TNS), a form of neuromodulation similar to vagus nerve stimulation (VNS), involving the non-invasive stimulation of the trigeminal sensory fibers, usually at the supraorbital or infraorbital nerve [81]. TNS has shown efficacy in drug-resistant epilepsy [82], major depression [83], migraine [84], and PTSD [85]. The similarity in the therapeutic effects between TNS and VNS can be attributed to their involvement of the same central structures: the nucleus tractus solitarius (NTS), locus coeruleus (LC), and dorsal raphe nucleus (dRN) [76].

The NTS is an important center in the regulation of visceral, cardiovascular, and respiratory functions, serving as an integrative center for the reflex control of the visceral motor function, as well as an intermediate stage for ascending pathways carrying visceral information to other brainstem or cerebral nuclei, such as the parabrachial nucleus, thalamus, hypothalamus, amygdala, insula, and prefrontal cortex (PFC) [86]. Ascending pathways originating from the NTS primarily project to the amygdala, but many interoceptive signals pass through the NTS to reach the insula [87]. The LC and dRN provide noradrenergic and serotonergic innervation to virtually the whole CNS, serving as key structures for autonomic function, arousal, the sleep–wake cycle, pain, anxiety, and mood [86]. Through these brainstem nuclei, trigeminal projections also reach the central encephalic structures involved in limbic and vegetative regulation, such as the hippocampus and amygdala [76].

Preclinical studies on TNS have demonstrated brain and systemic effects, such as variations in the interneuronal excitability in the brainstem [75] and increased neuronal proliferation [76] in the trigeminal, NTS, LC, dRN, dentate gyrus of the hippocampus, amygdala, endopiriform nucleus, and sensorimotor cortex. Variations in the EEG have also been observed [77]. Therefore, the trigeminal system is strategically involved in influencing the function and structure of the CNS, both at cortical and subcortical levels [76].

Clinical studies using TNS have demonstrated systemic and anti-inflammatory effects (Table 2).

In summary, the trigeminal nerve seems to play a central role in maintaining homeostasis and proper CNS function: sensory information collected by its terminal branches, both epicritic and protopathic, does not solely reach the sensory cortical centers, but becomes part of numerous pathways within the CNS that project to the autonomic nuclei and higher structures from a hierarchical and evolutionary perspective, such as the PFC and anterior cingulate cortex (ACC), which are essential to adaptive and predictive mechanisms involving cognitive, emotional, and behavioral components [78].

### 3.4. Trigeminal Innervation and the Trigemino-Vascular System in the Regulation of Cranial Neuroinflammation

The dense innervation that the trigeminal nerve sends to most cerebral blood vessels is referred to as the trigemino-vascular system and significantly contributes to the control of cerebrovascular tone [93,94]. Trigeminal fibers innervating the meninges and cerebral vessels mainly originate from V1 and consist of FNEs located at the arachnoid and pia mater, as well as around the cerebral arteries and dural venous sinuses [44].

Within the dura mater, trigeminal fibers, together with parasympathetic and sympathetic fibers [95], regulate the tropism of blood vessels: sympathetic fibers promote vasoconstriction, whereas parasympathetic fibers induce vasodilation [95].

Particularly, it has been demonstrated that the trigeminal nerve is capable of increasing the cerebral blood flow (CBF) through an antidromic pathway, trigemino-parasympathetic pathway, and central pathway [93] (Appendix C). Therefore, the trigeminal nerve and the trigemino-vascular system appear to play a crucial role in regulating cerebral metabolism, neuroinflammation, and controlling pathological cerebrovascular events (e.g., stroke, hemorrhages, traumatic brain injuries), as well as in the pathogenesis of migraine.

The activation of trigeminal nociceptive fibers leads to the release of CGRP, SP, and inflammatory cytokines into the perivascular space of the meninges [96]. These molecules can sensitize nerve endings, facilitating the establishment of a vicious circle: the more persistent the nociceptive stimulation, the more sensitized the nerve fibers become to new inputs, hence leading to a lowering of the nociceptive thresholds and to an enlargement of the receptive fields of the nerve endings [97]. The spread of this process at the level of the Gasser ganglion perpetuates peripheral sensitization and, over time, facilitates the establishment of central sensitization, which leads to increased pain perception and the chronification of pathologies such as migraine [97].

CGRP not only acts on meningeal blood vessels and nerve endings, but also on resident immune cells in the perivascular space, such as macrophages and mast cells [98]. These cells respond by activating and degranulating substances, such as histamine and inflammatory cytokines, which can further contribute to trigeminal fiber sensitization [99,100].

In case of neuroinflammation and sensitization, the activation of both endocranial meningeal fibers and exocranial fibers becomes relevant. Exocranial innervation by meningeal fibers appears to allow control of the intracranial metabolic, immune, and vascular function by relying not only on local biochemical information, but also on mechanical and thermal exteroception [101]. Exocranial fibers, therefore, represent a rapid and efficient system in this context to convey damage information, such as tissue injuries or a high temperature, and promote protective responses, such as increased local blood flow and leukocytes [48].

### 3.5. Interaction of Endocranial, Exocranial, Trigeminal, and Vascular Pathways in the Genesis of Cranial Pain

When searching the literature, it seems that the two main mechanisms underlying the processing of pericranial pain are neurogenic inflammation (at both the endocranial and exocranial levels) and sensitization of the trigeminal nuclei and DRG at C2–C4 [50,51,59].

Peripheral neurogenic inflammation exploits the antidromic pathway discussed earlier, responding to nociceptor activation with the release of neuropeptides and, subsequent, vasodilation and increased CBF. These molecules spread through antidromic conduction to the collateral branches of the trigeminal nerve, explaining two different mechanisms of pain development: (1) the endocranial origin of exocranial pain, which involve the activation of nociceptive fibers in the dura mater that spreads antidromically to the fibers that terminate outside the skull, leading to the activation of nearby somatic nociceptors through the local release of proinflammatory neuropeptides in the scalp [49,50,51,59]. This could explain why patients with migraine report cutaneous allodynia [102] and the sensation of cranial bones deforming, compressing, or breaking [103]. (2) The exocranial origin of endocranial pain, which involves the activation of nociceptors at the exocranial level (e.g., due to stimulation of pericranial muscles and arteries [104]) that spreads antidromically to the collaterals that terminate inside the skull, leading to increased meningeal blood flow, vasodilation, mast cell degranulation, and plasma extravasation [48,49,50,51,59,105]. An example of this phenomenon is occipital pain, where occipital muscle tension causing the compression of nerve fibers leads to the activation of the meningeal nociceptors [106].

There is also preliminary evidence on immune relationships between the dura and pia mater: in mice, neurogenic inflammation of the dura mater, particularly at the occipital level, can spread to the pia mater and, subsequently, to the brain parenchyma [70]. At the cerebellar level, this process could explain the presence of symptoms such as dizziness, motion sickness, and/or decreased motor coordination in patients with headaches [44].

Regarding the regulation of vascular function in a pathological context, trigeminal vascular control mechanisms appear to modulate ischemic and hypoxic phenomena that arise as a consequence of traumatic events (e.g., brain injury) and, if left uncontrolled, can further worsen the clinical picture in the presence of nerve tissue damage. To maintain cerebral blood flow, reliance is primarily placed on variations in vessel caliber and systemic arterial pressure [107].

In pathological states, alterations in these homeostatic mechanisms can facilitate the onset of neuronal damage and death, but these situations can be prevented by improving the cerebral blood flow, through cerebral vasodilation and/or increasing the mean systemic arterial pressure.

The trigeminal system, through the pathways described earlier, acts as a potent vasodilator, reducing cerebrovascular resistance in both physiological and pathological conditions [108,109]. In this regard, several authors report that the neurogenic control of CBF through the trigeminal nerve could be clinically exploited by using stimulation of the afferent trigeminal branches to induce vasodilation, restore cerebral autoregulation, and improve perfusion [93,110].

From a clinical perspective, the distribution of nerve fibers at the pericranial level can explain why pathologies such as migraine, in many cases, can be triggered in specific areas of the head (e.g., periorbital/temporal area) by the collateral exocranial branches of meningeal nociceptive fibers [50]. In this case, local neuroinflammation can be generated [59] due to increased densification of exocranial myofascial structures resulting from: cranial traumas [43,49]; cranio-cervical muscle tensions and contractions (including ATM dysfunctions) [44,111,112]; suture perforations in patients undergoing craniotomy in neurosurgical interventions [43,70].

This pain pattern can be maintained due to the subsequent central sensitization of the neurons present in the spinal trigeminal nucleus [43,51,59,102].

### 3.6. The Role of the Glymphatic System in Neuroinflammation of the Central Nervous System

Recent discoveries have shed light on the efflux of cerebrospinal fluid (CSF) through the glymphatic system, which facilitates drainage of the interstitial and perivascular spaces within the brain [113,114,115,116]. In addition to the well-known routes of CSF outflow through arachnoid villi, along the spinal and cranial nerves, and via the dural lymphatic capillaries, it has been found that CSF also passes along the perivascular spaces of small dural blood vessels that enter the cranial cavity through an extensive network of microscopic cranial canals [116]. These canals [117], coursing through the dura mater, connect the frontal, parietal, and occipital lobes to the marrow of their respective cranial bones [116].

This discovery suggests that the cranial marrow serves as a hematopoietic compartment responsive to CSF and, therefore, to the molecules it transports [116]. Considering that these canals enable bidirectional cellular trafficking between the cranial marrow, dura mater, and cerebral lobes [116,117], it is likely that sensitization of the exocranial fibers, in addition to what was mentioned earlier, may also alter the hematopoietic stem cells, and promote a more inflammatory state in leukocytes and their progenitors. As these microscopic channels provide direct access for the immune cells to the meninges, they may facilitate neuroinflammatory cascades within the trigemino-vascular, cervico-vascular, and cerebral parenchymal parts of the central nervous system, with broad implications for chronic neuroinflammatory neurological pathologies [43,116,117].

## 4. Discussion 

In the present perspective paper, the authors propose that mechanical, tactile, and thermal stimulation applied by osteopaths to the craniofacial region, specifically on cranial sutures, may improve painful dysfunctional syndromes and modulate neuroinflammation through endocranial effects on the trigeminal system, the meninges, the ANS, and the CNS. In particular, these effects are induced by the stimulation of the mechanoreceptors located in the exocranial neural pathways. This rationale could account for the physiological and clinical effects reported in the literature (Table 1 and Table 3), following the application of gentle and slow techniques that stimulate the C, CT, and Aδ fibers [67,118,119], which represents an approach typical in the OCF and OMT.

Supporting this claim are studies demonstrating clinical improvements in headache patients treated with local anesthetic or botulinum toxin injections in their peripheral nerves, pericranial trigger points [29,42,43,44], and particularly along the cranial sutures [49,50,51,59], densely innervated areas rich in C, CT, and Aδ afferents [50,70] that are highly sensitive to chemical, thermal, mechanical, nociceptive, and tactile stimuli [52]. The authors have even coined the term “Follow The Sutures” to describe their approach [59] 

Likewise, the slow and deep manual stimulation characteristic of OCF can non-invasively stimulate various trigeminal sensory fibers, including those within the supraorbital or infraorbital nerves [81]. The subsequent activation of the trigeminal nuclei may promote neuro-immune rebalancing, starting with ANS activity, through the homeostatic regulatory pathways identified in the literature [76,77,78,81], and extending to the CNS, with implications for central and peripheral nociceptive sensitization, neuroinflammation, and pain [118].

Further exploring the stimulation of the mechanotransduction pathways in the pericranial and exocranial regions, OCF may modulate the trigemino-vascular system by targeting specific mechanoreceptors, including the aforementioned Piezo2, which are indeed activated by tactile stimuli and are expressed, for instance, in Merkel discs [129,130].

In recent years, several studies have revealed systemic effects resulting from the stimulation of Merkel discs, which are abundantly present in regions of the face sensitive to touch and are associated with hair and scalp areas [131], which form units known as Merkel nerve complexes (MNCs) involved in the neuro-immune response following nerve stimulation and are endowed with neuroendocrine activity. MNCs can release various substances, including glutamate, SP, CGRP, vasoactive intestinal peptide, the same molecules implicated in the trigeminal nociceptive network, capable of altering local and systemic hemodynamics and muscle contraction, as well as activating the interoceptive network and the CAN, thus acting as a bridge between exteroceptive and interoceptive communication pathways [52,132,133,134].

Regarding TRP channels, particularly present in C and Aδ fibers at the pericranial level [59], specific studies on their activation by touch are lacking. However, given several pieces of evidence on mechanotransduction pathway activation through manual stimulation linked to TRP channels (Table 4) [135,136], it is plausible that OCF may also stimulate these channels.

OCF might indirectly stimulate TRP channels through the release of endocannabinoids, which would have significant regulatory effects on neuroinflammation and chronic pain: TRP channels demonstrate high sensitivity to these molecules. Some studies have shown that OMT can induce the release of anandamide in healthy subjects and N-palmitoylethanolamide (PEA) in individuals with chronic lower back pain [137,138]. Anandamide is a potent endogenous agonist of the TRPV1 channel, while PEA primarily activates it indirectly by inhibiting anandamide degradation [58].

In support of the above statement, endocannabinoid system stimulation has shown promise as a strategy for individuals with headaches, as it activates both CB1 and CB2 cannabinoid receptors, as well as certain TRP channels, leading to reduced neuroinflammation, pain, and improved quality of life [58,139].

It is noteworthy that endocannabinoid release does not seem to occur in subjects treated with a sham protocol [137,138].

**Table 4:** Mechanotransduction effects observed in studies on touch, manual therapies, or using “realistic” laboratory models (i.e., using stimuli that are replicable in clinical practice).

References	Type of Study	Mechanotransduction Effects
Evanko, 2009 [140]	Commentary	Influence mediated by the activation of membrane integrins, the prototypical mechanosensitive proteins, on growth, alignment, and metabolic and secretory activity of keratinocytes and fibroblasts
Gehlsen et al., 1999 [141]	Randomized control trial on augmented soft tissue mobilization therapy, a rat model	
Pohl, 2010 [142]	Observational study (pre-post) about manual therapy	
Silver et al., 2003 [143]	Review	
Hick et al., 2012 [144]	In vitro model of myofascial release after repetitive motion strain applied to fibroblasts and myoblasts	Increased expression of nicotinic acetylcholine receptors (nAChR) in myoblasts
Hick et al., 2014 [145]	In vitro model of myofascial release after repetitive motion strain applied to fibroblasts and myoblasts	
Zein-Hammoud and Standley, 2015 [146]	Review about in vitro models of myofascial release and counterstrain	
Crane et al., 2012 [147]	Randomized controlled trial about massage therapy vs. control (no therapy)	Increased expression and activation of mechanotransduction pathways, e.g., extracellular signal-regulated protein kinases 1 and 2 (ERK1/2), focal adhesion kinases (FAKs), and peroxisome proliferation factor (PGC-1α), relevant to cell metabolism
Miller et al., 2018 [148]	Rat model of massage therapy after skeletal muscle atrophy	
Wan et al., 2019 [149]	Rat model of massage therapy after skeletal muscle atrophy	Increased expression of the protein Akt, involved in cell survival
Cao et al., 2013 [150]	In vitro model of myofascial release applied to fibroblasts	Variations in the secretion of inflammatory cytokines, e.g., interleukin-1β (IL-1β), IL-3, IL-6, prostaglandin-E2, angiotensin II, platelet-derived growth factor (PDGF), granulocyte colony-stimulating factor (GCSF), and macrophage-derived chemokine (MDC), as well as in the production of antioxidant substances (SOD) and the expression of inflammatory genes, such as nuclear factor κB (NF-κB), in muscle cells and fibroblasts
Crane et al., 2012 [147]	Randomized controlled trial about massage therapy vs. control (no therapy)	
Eagan et al., 2007 [151]	In vitro model of manual medicine treatment applied to fibroblasts	
Silver et al., 2003 [143]	Review	
Zein-Hammoud and Standley, 2015 [146]	Review about in vitro models of myofascial release and counterstrain	
Zhang et al., 2016 [152]	In vitro model of rolling manipulation in traditional Chinese medicine applied to human skeletal muscle cells	

### 4.1. Limitations

While the present perspective paper on exo- and endocranial communication pathways, from the mechanosensitive afferents to neuroimmune regulation effects or their role in pain genesis [48,49,50,51,59,104,105,106], may constitute a plausible testable scientific rationale for OCF, at the time of writing, the scientific evidence supporting OCF remains scarce and incomplete, and even more so, regarding the evidence on the underlying mechanisms.

For instance, McPartland’s reflections highlight the scarcity of studies examining the relationship between OMT (or manual therapies more broadly) and cannabinoids [153], in contrast to numerous other fields, such as pharmacology, nutrition, and nutraceutical integration. As a consequence, clinicians, researchers, decisionmakers, and stakeholders rely on dated studies with weak methodologies and outdated investigative tools for taking decisions regarding increasingly relevant topics, such as neuroinflammation management in chronic diseases like migraine [154,155], where manual therapies could potentially be beneficial [30].

This observation can easily extend to various other areas, such as TRP channels. However, almost every article discussing these channels presents them as sensors responsive to chemical, thermal, and mechanical stimuli, and thus are fundamental to many sensory processes; for instance, the abstract of a 2008 article recites: “As such, TRP channels play a crucial role in many mammalian senses, including touch, taste, and smell” [156]. Similarly, a 2021 article claims: “They are involved in critical roles in sensory physiology such as vision, smell, hearing, taste, and touch” [60]. Different basic science studies have reported a significant improvement in neurotransmission and spatial memory in aged rat brains [157,158]. Despite various tools to detect and manipulate mechanoreceptor information encoding being available, studies conducted in clinical settings and, most importantly, in humans are rarely cited [159].

Moreover, in recent years, studies on manual therapies have particularly focused on detecting “physiological” effects, such as changes in heart rate variability (HRV) [120,160], a biomarker considered by many authors as the “gateway” to studying ANS functionality [161] and the subsequent neuroimmune and endocrine regulatory functions [162], or on identifying “neurocentric” effects at the cerebral level in order to understand the physiological and behavioral regulatory networks elicited by manual touch [126,127,163,164]. In addition, other authors have explored the OCF rationale referring to ANS activity related to cranial hemoliquorodynamics [165,166] and have associated the amplitude and timing of blood pressure variability with respiration (i.e., Traube–Hering–Mayer oscillations) [167,168,169].

Despite their usefulness, the result is a lack of direct studies examining the specific mechanisms underlying the manifestation of these effects, which poses the risk of not fully understanding whether these effects indeed stem from the touch itself or from other factors, such as a placebo [170].

In the absence of such knowledge, it becomes more challenging for clinicians to choose the most appropriate treatment path for a patient and for stakeholders to decide how to organize healthcare resources [154].

The present paper focuses on the current knowledge in the fields of neurophysiology and mechanobiology to propose an evidence-informed rationale for OCF. Despite the proposed rationale being centered on biological elements, the authors highly recommend that researchers, academics, and clinicians inform the perspective through neurocognitive and psychosocial sciences, i.e., implementing the (en)active inference and neuroaesthetic theoretical frameworks [171]. The future research agenda must follow a roadmap to address the limitations of the presented rationale, also addressing the lack of randomized clinical trials using placebo treatments in the control group.

### 4.2. Future Directions

This perspective paper has been developed by analyzing the current knowledge concerning the tactile stimulation of exocranial receptors, the related endocranial and systemic effects, aiming to consider their implications in treating various pain and dysfunctional conditions and to establish a potential scientific rationale for OCF. A new theoretical model based on scientific evidence could foster the development of novel approaches for research and clinical practice in the field of OCF and, more broadly, in manual therapies and osteopathy.

For such a new theoretical model to be valid and reliable, it must be testable and, therefore, falsifiable. Hence, considering the sections presented earlier, the authors call for specific research to fully understand the effects of osteopathic touch in OCF on exocranial innervation, particularly at the level of sutures. They propose key points in the rationale to be analyzed through future studies (Table 5).

Thoroughly investigating these individual steps using state-of-the-art medical research technologies, revisiting studies that characterized CT pathways or the tactile activation of Piezo2 channels [52,129], would provide a more comprehensive and defined understanding on the potential usefulness and efficacy of manual therapies, particularly OCF. Moreover, researchers should better investigate the osteopathic management of deformational plagiocephaly described in a recently published review, as linked to the sensitization of the mechanoreceptors in the upper neck–head regions, and related to the brainstem and trigeminal system sensitization [172]. Positive research outcomes would aid clinicians in better advising patients on this form of therapy and evaluating its integration with other treatment protocols, a practice currently challenging due to the limited scientific evidence available. To the best of our knowledge, even if many healthcare interventions may be beneficial, the available high-quality reviews report that the majority of therapeutic procedures in medicine do not have high-quality scientific evidence supporting their benefits [173]. This problem might be remedied by high-quality studies in healthcare-critical areas, including osteopathic care and OCF.

## 5. Conclusions

In conclusion, this perspective paper has explored several key aspects related to craniofacial tactile mechanosensation and its implications in exo- and endocranial communication. The distribution of touch-sensitive mechanoreceptors in the pericranial region, particularly concentrated within sutures, forms a crucial network for mediating exo- and endocranial connections. The paper has also highlighted the systemic responses triggered by exo-and endocranial communication, facilitated by the trigeminal system and the CAN. Understanding these interactions is vital in comprehending the regulation of cranial neuroinflammation. Furthermore, the trigeminal innervation and the trigemino-vascular system play significant roles in the genesis of cranial pain. The intricate interplay of endo- and exocranial, trigeminal, and vascular communication pathways contribute to pain manifestation. Additionally, recent discoveries regarding the role of the glymphatic system in neuroinflammation further enrich our understanding of cranial pathophysiology.

The interconnection of these topics provides a comprehensive picture of the complex network of cranial physiology, particularly regarding pain and inflammatory processes. These insights open new possibilities for research and clinical practice in manual therapies, including OCF. By continuing to investigate and validate these concepts through rigorous scientific studies, we can advance our understanding of the benefits of manual touch and OCF in regard to various cranial conditions. Furthermore, this deeper comprehension could lead to more targeted and effective therapeutic approaches for patients suffering from cranial pain and dysfunction. Overall, the integration of these interconnected aspects may pave the way for innovative and evidence-based strategies in osteopathy and manual therapies, ultimately benefiting patient care and overall wellbeing.

## Figures and Tables

**Table 1:** Potential effect of OCF and/or OMT through cranial techniques.

References	Type of Study	Pathologies
Biondi, 2005 [29]	Review about chiropractic, osteopathic, physical therapy, or massage interventions	Headache and migraine
Cerritelli et al., 2015 [30]	A 3-armed randomized controlled trial (OMT + medical therapy vs. sham + medical therapy vs. medical therapy)	
Biondi, 2005 [29]	Review about chiropractic, osteopathic, physical therapy, or massage interventions	Facial pain
Detoni et al., 2022 [31]	Randomized controlled study about OMT vs. molar shim	Temporo-mandibular joint dysfunction (TJD)
Easterbrook et al., 2019 [32]	Commentary with video to show osteopathic approach	
Lancaster and Crow, 2006 [33]	Case report	Bell’s paralysis
Volokitin et al., 2020 [34]	Case report	
Karp et al., 2019 [35]	Retrospective case review about facial rehabilitation	Facial nerve dysfunctions (and other conditions related to exocranial nerve dysfunction)
Khan et al., 2022 [36]	Systematic review about massage, exercise, and facial rehabilitation	
Zurowska et al., 2017 [23]	Systematic review about compression of the fourth ventricle	Blood pressure, sleep onset, tension-type headache
Haller et al., 2020 [27]	Systematic review and meta-analysis of randomized controlled trials about craniosacral therapy	Chronic pain
Parravicini and Ghiringhelli, 2021 [25]	Case report	Whiplash-associated disorder
Dickerson et al., 2022 [21]	Preliminary study about cranial osteopathic manipulative medicine	Brain injury recovery
Terrell et al., 2022 [26]	Randomized controlled trial about OMT vs. osteopathic cranial manipulative medicine	Parkinsonian gait

**Table 2:** Summary of effects that can be induced by trigeminal nerve stimulation (TNS).

References	Type of Study	Effects
Suzuki et al., 2020 [88]	Uncontrolled trial about electroacupuncture	Increase in blood flow and oxygenation in the prefrontal cortex (PFC), reduction in heart rate, and an increase in heart rate variability (HRV) through electroacupuncture of the ophthalmic branch of the trigeminal nerve.
Waki et al., 2017 [89]	Randomized controlled trial about electroacupuncture vs. control (no therapy)	
Magis et al., 2017 [90]	Uncontrolled trial about external trigeminal nerve stimulation	Increased cerebral metabolism in the orbitofrontal cortex (OFC) and rostral anterior cingulate cortex (rACC) in individuals suffering from migraines.
Ritland et al., 2022 [91]	Randomized controlled trial about trigeminal nerve stimulation vs. sham	Modulation of neurochemical concentrations within the central nervous system, particularly a decrease in total creatine levels in the dorsolateral prefrontal cortex (dlPFC).
Chiluwal et al., 2017 [92]	Controlled study about trigeminal nerve stimulation after severe induced traumatic brain injury	Anti-inflammatory effect in the brain observed in an animal model with traumatic brain injury.

**Table 3:** Physiological effects induced by OCF and/or OMT through cranial techniques.

References	Type of Study	Effects
Bove, 2013 [42]	Commentary about physical therapies (e.g., spinal manipulation, massage)	Decreased afferent nociceptive input
Cerritelli et al., 2020 [120]	Randomized controlled trial about OMT vs. sham	Influence on insula, anterior cingulate cortex (ACC), and brain networks related to interoception
Casals-Gutiérrez and Abbey, 2019 [121]	Systematic review that included touch interventions	
Jäkel and von Hauenschild, 2011 [122]	Systematic review about cranial osteopathic manipulative medicine	Autonomic nervous system (ANS) modulation, regulation between parasympathetic and sympathetic activity, as revealed by heart rate variability (HRV) analysis
Curi et al., 2018 [123]	Controlled clinical trial: compression of the fourth ventricle in hypertensive and normotensive people	
Bayo-Tallón et al., 2019 [124]	Randomized controlled trial about manual cranial therapy vs. massage therapy	
Ponzo et al., 2018 [125]	Crossover study: OMT vs. muscle stretching vs. soft touch	Enhanced corticospinal excitability
Tamburella et al., 2019 [126]	Randomized controlled trial about OMT vs. sham	Changes in resting cerebral perfusion
Cerritelli et al., 2021 [127]	Randomized controlled trial about OMT vs. sham	Positive affect of the pain network
Dugailly et al., 2014 [128]	Randomized controlled trial about OMT vs. control (no therapy)	Reduced anxiety symptoms and increased overall body perception in female subjects
Miana et al., 2013 [16]	Randomized controlled trial: compression of the fourth ventricle vs. sham vs. control (no therapy)	Increased alpha band absolute power in quantitative electroencephalography
Fornari et al., 2017 [24]	Randomized controlled trial about OMT vs. sham	Reduced mental stress
Abenavoli et al., 2020 [19]	Randomized controlled trial: compression of the fourth ventricle vs. sham vs. control (no therapy)	Increased salivary alpha amylase activity

**Table 5:** Key points in the proposed rationale to be examined in future studies (briefly outlining the study types).

Key Points/Objectives	Type of Studies
Whether manual touch in general and OCF, in particular, activate exocranial nerve pathways.	Basic science neurophysiological studies following manual stimulation, that is, a non-specific tactile stimulus and a tactile stimulus that resembles the kind of touch used in OCF. The study must be performed in both healthy and symptomatic populations.
Whether activation of exocranial nerve pathways through manual touch leads to the activation of endocranial nerve pathways.	Basic science neurophysiological studies following manual stimulation, that is, a non-specific tactile stimulus and a tactile stimulus that resembles the kind of touch used in OCF. The study must be performed in both healthy and symptomatic populations.
Whether manual touch in general and OCF, in particular, activate specific TRP channels present in exocranial nerve pathways.	Basic science studies following manual stimulation, that is, a non-specific tactile stimulus and a tactile stimulus that resembles the kind of touch used in OCF. The study must be performed in both healthy and symptomatic populations.
Whether TRP channel activation in the cranial area is indirectly influenced by factors induced by touch, e.g., the aforementioned endocannabinoids.	Basic science studies following manual stimulation, that is, a non-specific tactile stimulus and a tactile stimulus that resembles the kind of touch used in OCF. A literature analysis should guide the choice of chemical mediator (e.g., endocannabinoids) to observe and block activation. The study must be performed in both healthy and symptomatic populations.
Whether TRP channel activation induced by OCF shows neuro-immune modulation effects.	Randomized controlled trials, OCF vs. OCF-no-TRP (see below) vs. sham, to evaluate a specific neuro-immune modulatory effect (e.g., reduced inflammation) in both healthy and symptomatic populations. In the group OCF-no-TRP, the researchers should use pharmacological agents to inhibit TRP activation.
Whether manual touch in general and OCF, in particular, influence the trigemino-vascular system.	Basic science studies following manual stimulation, that is, a non-specific tactile stimulus and a tactile stimulus that resembles the kind of touch used in OCF. The study must be performed in both healthy and symptomatic populations.
Whether manual touch in general and OCF, in particular, affect the flow of the glymphatic system.	Randomized controlled trials, OCF vs. sham, to evaluate the specific effect in both healthy and symptomatic populations.
Whether manual touch in general and OCF, in particular, act on the cranial marrow, influencing hematopoietic stem cells, conditioning the development of leukocytes and their progenitors, and affecting the release of pro- or anti-inflammatory molecules.	Randomized controlled trials, OCF vs. sham, to evaluate the specific effect in both healthy and symptomatic populations.
Whether local endocranial neuroinflammation can result in tissue distortions at the myofascial and cutaneous levels and whether these alterations can be detected and recognized through osteopathic palpation.	(1) Basic science and pharmacological studies to induce mild neuroinflammation and ascertain its effects. (2) Diagnostic studies that compare osteopathic palpation with an appropriate gold standard on subjects with induced neuroinflammation to assess myofascial and cutaneous tissue distortions.

## Data Availability

Not applicable.

## Appendix A

The pathways through which the nerves pass from the external to the internal cranial vault, and vice versa, are as follows: (1) cranial vault sutures, which represent the most important route for sensory and nociceptive fibers. The primarily involved sutures are the coronal, squamous, sagittal, and lambdoid sutures. Among these, the coronal and squamous sutures exhibit the highest innervation density. (2) Emissary canals: the channels present in the cranial vault bones, through which emissary veins drain blood from the diploic veins in bone and dural sinuses. Nerve fibers pass bidirectionally between the scalp and dura mater in these canals. (3) Cervico-occipital canals: the greater and lesser occipital nerves, originating from C2, form bundles of large and medium-sized fibers, which, accompanying blood vessels, travel on the surface and within muscles, such as the trapezius, splenius, and semispinalis capitis. After their course through the neck muscles, many fibers penetrate the skull through five pathways: (I) a medium-sized canal formed by the junction of the occipital bone and petrous part of the temporal bone; (II) the emissary canals in the occipital condyle; (III) the hypoglossal canal; (IV) the foramen magnum; (V) the jugular foramen.

## Appendix B

The dural nerves follow three main pathways to converge into the trigeminal nerve and one pathway that, through the spinal nerves at C2–C3, leads to the spinal cord: (1) the anterior pathway, which originates in the anterior cranial fossa, this pathway follows the anterior and posterior ethmoidal nerves to connect with V1. These nerves, along with the supraorbital and supratrochlear nerves, innervate not only the dura mater in the anterior cranial fossa, but also the periosteum in the frontal and partly parietal bones. (2) The lateral pathway, which innervates the middle cranial fossa and follows the meningeal branches of V2 and V3, running together with the branches in the middle meningeal artery. These fibers are connected with nerves that innervate the exocranial part, particularly the zygomaticotemporal nerve (a branch of V2) at the temple and the auriculotemporal nerve (a branch of V3). (3) The posterior-superior pathway, which transports the fibers of the transverse sinus, torcular, tentorium, and posterior part of the falx to V1, following the tentorial nerve. (4) The posterior-inferior pathway: from the dura mater below the tentorium, fibers travel to the dorsal root ganglia at the C2–C3 level. From here, the fibers enter the spinal cord at the level of the laminae I-V and connect to wide dynamic range (WDR) interneurons in C2–C4. This pathway innervates not only the dura mater in this specific cranial area, but also the cutaneous, muscular, and fascial areas surrounding the ears and occipital region.

## Appendix C

The trigeminal nerve has been shown to increase cerebral blood flow through three pathways: (1) the antidromic pathway, stimulation of the sensory fibers in the trigeminal nerve, even at the exocranial level, activates an antidromic pathway that releases vasoactive peptides (calcitonin gene-related peptide and substance P) in the nerve-innervated territories, causing cerebral vasodilation and increased cerebral blood flow. (2) The trigemino-parasympathetic pathway: the afferent branch of this pathway mainly involves V3, while the efferent branch comprises parasympathetic fibers that reach the periphery via the facial nerve. Stimulation of trigeminal fibers leads to parasympathetic vasodilation at the cerebral level, mediated by the connections with the facial nerve and the pterygopalatine ganglion. (3) The central pathway, i.e., trigeminal afferents, upon reaching the trigeminal nuclei within the central nervous system, project to various nuclei, including the rostral ventrolateral medulla.
